# MiR-455-3p inhibits the degenerate process of chondrogenic differentiation through modification of DNA methylation

**DOI:** 10.1038/s41419-018-0565-2

**Published:** 2018-05-10

**Authors:** Hao Sun, Xiaoyi Zhao, Chengyun Zhang, Ziji Zhang, Jiayong Lun, Weiming Liao, Zhiqi Zhang

**Affiliations:** Department of Joint Surgery, First Affiliated Hospital of Sun Yat-Sen University, 510080 Guangzhou, Guangdong China

## Abstract

The aim of this work was to determine whether miR-455-3p regulates DNA methylation during chondrogenic differentiation of hMSCs. The expression of miR-455-3p and de novo methyltransferase DNMT3A was assessed in micromass culture of hBMSCs, which induced chondrogenic differentiation in vitro, and in E16.5 mice in vivo. A luciferase reporter assay was used to confirm whether miR-455-3p directly targets DNMT3A by interaction with the 3′-UTR. Using an Illumina Infinium Methylation EPIC microarray, genome-wide DNA methylation of hBMSCs with or without overexpressed miR-455-3p was examined for 28 days during induced chondrogenic differentiation. Here, we showed that miR-455-3p was more expressed during the middle stage of hBMSC chondrogenic differentiation, and less expressed in the late stage. DNMT3A was less expressed in the middle stage and more expressed in the late stage, and was also more expressed in the palms of miR-455-3p deletion mice compared to those of wild-type mice. The luciferase reporter assay demonstrated that miR-455-3p directly targets DNMT3A 3′-UTR. miR-455-3p overexpression inhibits the degenerate process during chondrogenic differentiation, while deletion of miR-455-3p in mice accelerated cartilage degeneration. Genome-wide DNA methylation analysis showed miR-455-3p overexpression regulates DNA methylation of cartilage-specific genes. GO analysis revealed PI3K-Akt signaling pathway was most hypomethylated. Our data show that miR-455-3p can regulate hMSC chondrogenic differentiation by affecting DNA methylation. Overexpression of miR-455-3p and DNA methylation inhibitors can thus potentially be utilized to optimize chondrogenic differentiation.

## Introduction

It is widely accepted that DNA methylation occurs at the sites of CpG dinucleotides, plays a critical role in the regulation of gene expression, and is involved in a variety of biological processes^1,2^. DNA methylation is regulated by DNA methyltransferases (DNMTs), which promote methylation, and by the ten-eleven translocation (TET) family of 5mC dioxygenases, which are involved in demethylation. There are three mainly active DNMT enzymes, namely *DNMT1, DNMT3A*, and *DNMT3B*^3^; *DNMT1* is the major maintenance methyltransferase while *DNMT3A* and *DNMT3B* are primarily de novo methyltransferases^4^.

The effect of DNA methylation in osteoarthritis (OA) pathophysiology is becoming increasingly evident. OA progress is accompanied by methylation changes to cartilage catabolic and developmentally associated genes such as *MMP13*, *SOX9*, *ADAMTS4*^5–9^. DNA has been shown to be differentially methylated between normal and OA cartilage^5^. For example, the enhancer region of NF-κB at −5.8 kb was significantly demethylated in OA cartilage compared with the same region in control samples, leading to the activation of inducible nitric oxide synthase to start or accelerate OA process^6^. Recent studies showed that *MMP13*^7^ and *ADAMTS4*^8^ decreased methylation and *SOX9*^9^ increased methylation in OA, and the gene expression is inversely related to methylation. Moreover, increased DNA methylation due to age and oxidative stress impaired the differentiation capacity of hMSCs^10^, and DNA methyltransferase inhibitors promoted the differentiation of stem cells by repressing DNA methylation^11^.

MicroRNAs (miRNAs) are short (~22 nt), non-coding, single-stranded RNAs that function as post-transcriptional regulators. Specifically, miRNAs silence gene expression by binding to the 3′-untranslated region (3′-UTR) of specific target mRNAs, thereby promoting their degradation or inhibiting their translation^12^. Previously, we found high expression levels of miR-455-3p in human adipose-derived stem cells (hADSCs) during chondrogenic differentiation^13^. We subsequently demonstrated that miR-455-3p plays a role in regulating OA and the chondrogenic differentiation of hMSCs^14–16^.

Recent studies have shown that miR-455-3p regulates certain pathological processes, such as cancer^17^. Our previous study showed that miR-455-3p functions as an activator for early chondrogenic differentiation of ATDC5 cells, and directly targets and inhibits the expression of Runt-related transcription factor 2 (RUNX2)^15^. We also demonstrated that miR-455-3p promotes chondrogenic differentiation by suppressing the expression of *HDAC2* and *HDAC8*, thereby maintaining an appropriate level of histone H3 acetylation at the* COL2A1* promoter to promote the production of type II collagen^14^. However, whether miR-455-3p regulates cartilage development and degeneration by adjusting DNA methylation level is not clear. Using miRNA target prediction algorithms, we found that miR-455-3p has a potential target in* DNMT3A*, which does regulate DNA methylation. Thus, we hypothesized that miR-455-3p plays an important role in hMSC chondrogenic differentiation by regulating the ability of *DNMT3A* to modulate DNA methylation. In this study, we investigated the role of miR-455-3p in the process of hMSC chondrogenic differentiation by characterizing its effect on DNA methylation of cartilage-specific genes and pathways.

## Results

### Expression pattern of miR-455-3p and DNMT3A during chondrogenic differentiation of hBMSCs

**Fig. 1:**
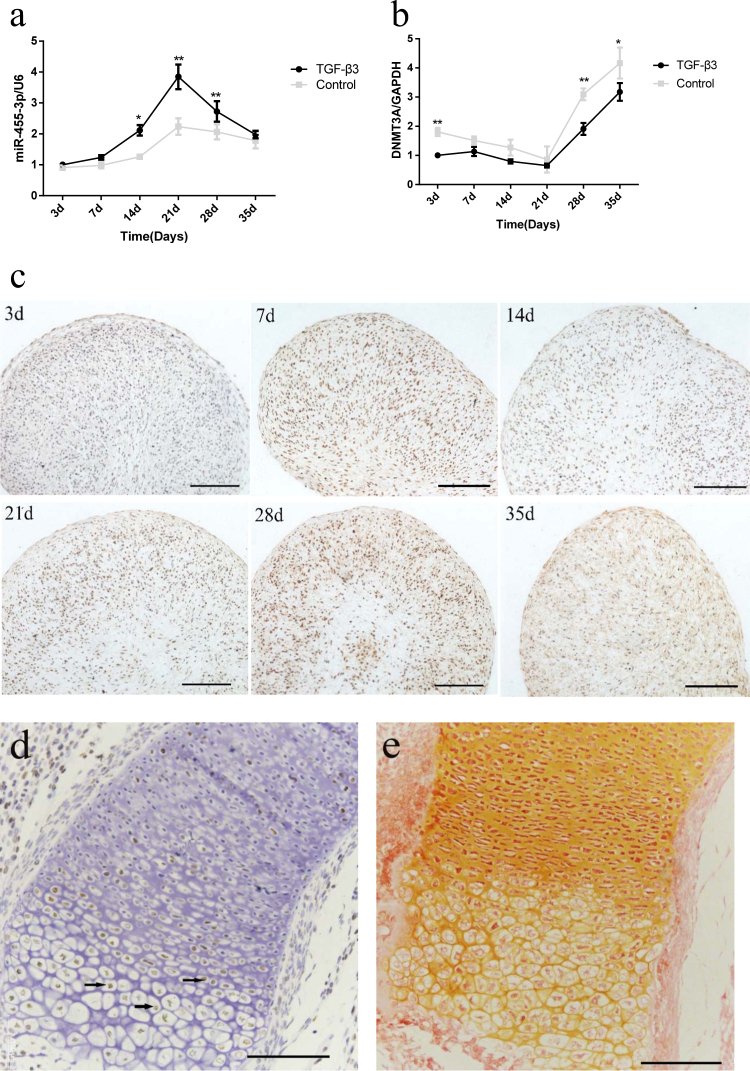
Analysis of miR-455-3p and DNMT3A expression during chondrogenesis. hBMSCs cultured in chondrogenic differentiation medium for 0, 7, 14, 21, 28, and 35 days. The expression of miR-455-3p (**a**), DNMT3A (**b**) were detected by qRT-PCR. hBMSCs cultured without TGF-β3 at corresponding time points served as negative controls. U6 and GAPDH expression levels were measured and used as internal controls for microRNA and mRNA expression, respectively. hBMSCs cultured with TGF-β3 were stained with immunohistochemistry of DNMT3A (**c**). The humerus in mouse embryos at E16.5 were immunostained with DNMT3A (**d**) and safranin O (**e**), scale bar, 100 μm. Data are presented as means ± SD of the results obtained from triplicate samples. *P < 0.05; **P < 0.001 vs. negative controls, respectively

To characterize the expression patterns of miR-455-3p and DNMT3A during chondrogenic differentiation, hBMSCs were induced to differentiate into chondrocytes in micromass culture in vitro. Safranin O staining at days 21 and 35 showed successful chondrogenic differentiation of hBMSCs (Supplementary Figure 1). The expression of miR-455-3p increased rapidly in hBMSC chondrogenic differentiation beginning on day 7, peaked at 21 days, and was followed by a marked decrease from days 28 to 35 (Fig. 1a). Meanwhile, the opposite trend was evident in *DNMT3A*expression, which descended from days 3 to 21 and then rapidly increased from days 28 to 35 (Fig. 1b). Immunohistochemistry analysis of *DNMT3A* (Fig. 1c) also showed a similar trend to RT-PCR, suggesting that miR-455-3p may affect the expression of DNMT3A.

Our previous study^14^ revealed that while moderate and high levels of miR-455-3p expression were detected in proliferating and prehypertrophic chondrocytes, little to no miR-455-3p expression was observed in hypertrophic chondrocytes in vivo by in situ hybridization in E16.5 mouse limbs. Therefore, we performed DNMT3A staining in E16.5 mouse limbs to characterize the expression patterns of DNMT3A. The result showed while low levels of DNMT3A expression were detected in proliferating chondrocytes, moderate and high levels of DNMT3A expression was observed in prehypertrophic and hypertrophic chondrocytes (Fig. 1d, e). These contrary expression patterns between miR-455-3p and DNMT3A indicated the co-regulation of chondrogenesis.

### MiR-455-3p regulates the expression of DNMT3A in vitro and in vivo

**Fig. 2:**
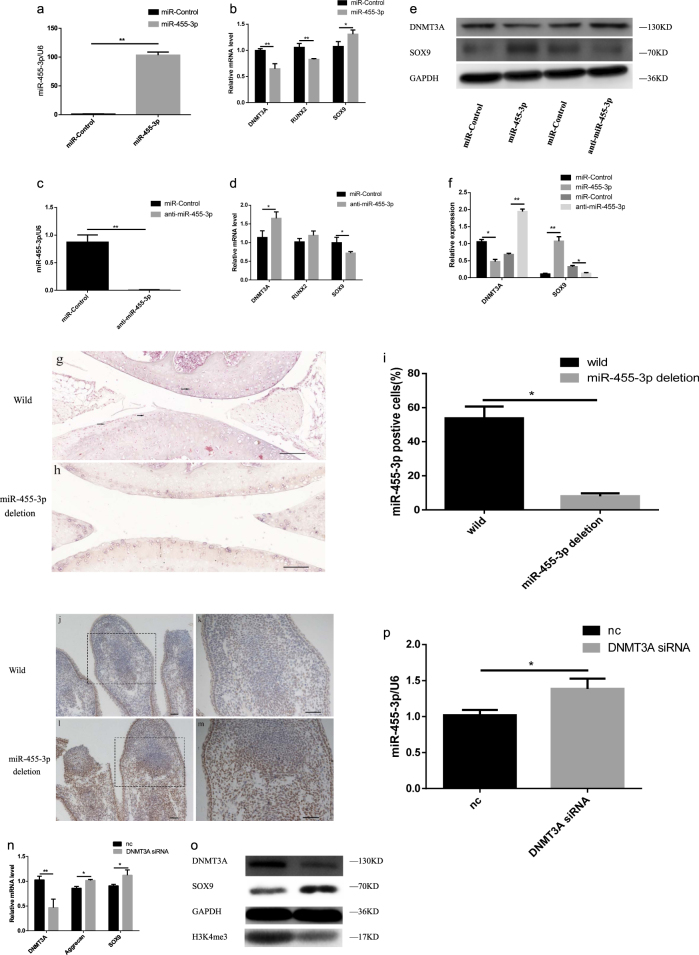
miR-455-3p inhibits the expression of DNMT3A, and DNMT3A also modulated expression of miR-455-3p. Normal chondrocytes were transfected with miR-455-3p mimic, a non-specific control (NC) mimic, miR-455-3p inhibitor, NC inhibitor. The effect of overexpressed (**a**, **b**) or inhibited (**c**, **d**) miR-455-3p on DNMT3A, RUNX2, and SOX9 were detected by RT-PCR. Western blot analysis of DNMT3A and SOX9 expression levels (**e**), and quantification (**f**). Situ hybridization of knee cartilage (**g**, **h**) showed the miR-455-3p expression in miR-455-3p deletion mice (**h**) was repressed compared with wild mice (**g**), scale bar, 100 μm. The expression of DNMT3A in palm of wild mice (**j**, **k**) and miR-455-3p deletion mice (**l**, **m**), scale bar, 100 μm. DNMT3A siRNA was transfected into normal chondrocytes, then RT-PCR and western blot were used to detect the expression of DNMT3A, Aggrecan, Sox9, H3K4me3 (**n**, **o**) and miR-455-3p (**p**). Data are presented as means ± SD of the results of three independent experiments. *P < 0.05, **P < 0.001. GAPDH expression levels were measured and used as internal controls. The experiment was performed in triplicate and a representative image is shown

To further investigate whether miR-455-3p regulates *DNMT3A* expression in vitro and in vivo, we inhibited and overexpressed miR-455-3p. The chondrocytes isolated from normal cartilage were transfected with either miR-455-3p or anti-miR-455-3p (Fig. 2a, c). The expression levels of* DNMT3A* and *SOX9* were assessed by qRT-PCR and western blotting. Treatment with miR-455-3p resulted in significantly decreased mRNA expression of DNMT3A and an increase in *SOX9* expression (Fig. 2b, e, f). Meanwhile, inhibition of miR-455-3p resulted in a significant increase in the expression of *DNMT3A* and a decrease in *SOX9* expression (Fig. 2d−f).

We constructed miR-455-3p deletion mice and proved the successful deletion. We compared miR-455-3p expression between 6-month wild-type mice and homozygous mice by in situ hybridization. The results showed miR-455-3p were significantly decreased on cartilage (Fig. 2g−i), bone (Supplementary Figure 2a, b, e), and meniscus (Supplementary Figure 2c, d, e). For the in vivo study, the embryos with wild-type, heterozygous, and homozygous genotypes were obtained through the mating of heterozygous male and female mice. We isolated the forelimbs of mice at embryonic stage 16.5 (E16.5) and characterized the expression patterns of DNMT3A. Notably, we observed high DNMT3A expression levels in palms of miR-455-3p deletion mice (Fig. 2l, m), while low levels were detected in wild-type mice (Fig. 2j, k). These results suggest that miR-455-3p may affect the expression of DNMT3A and regulate cartilage metabolism.

### DNMT3A modulated expression of endogenous miR-455-3p in normal chondrocytes

To assess whether DNMT3A modulated expression of miR-455-3p in normal chondrocytes, we transfected with *DNMT3A* siRNA. The expression levels of miR-455-3p were assessed by qRT-PCR, and the expression of *DNMT3A* was assessed by qRT-PCR and western blotting. Treatment with *DNMT3A* siRNA resulted in significantly decreased expression of *DNMT3A*and *H3K4me3* (Fig. 2n, o), and increased expression of miR-455-3p, aggrecan, and SOX9 (Fig. 2n−p).

### MiR-455-3p directly targets DNMT3A by interaction with the 3′-UTR

**Fig. 3:**
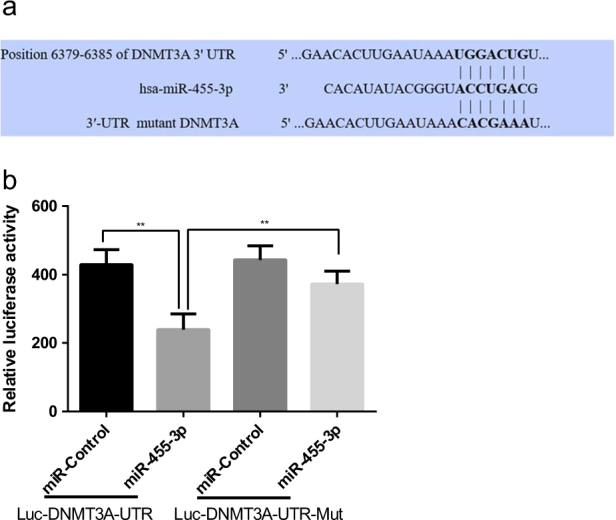
miR-455-3p directly targets DNMT3A. Alignment of the nucleotide sequence of miR-455-3p, 3′-UTR DNMT3A, and 3′-UTR mutant DNMT3A mRNA (**a**). A luciferase reporter carrying the 3′-UTR of DNMT3A or mutant DNMT3A in which the binding site of miR-455-3p was mutated (Luc-DNMT3A-UTR-mut) was introduced into 293T cells along with negative miR-control (miR-Control), miR-455-3p, respectively. The cells were harvested 48 h later for luciferase assays (**b**). Data are presented as means ± SD of the results of three independent experiments. *P < 0.05, **P < 0.001

TargetScan analysis identified *DNMT3A* as a potential target of hsa-miR-455-3p (Fig. 3a). We, therefore, utilized a luciferase reporter assay to further examine whether the *DNMT3A* 3′-UTR contained a sequence capable of interacting with miR-455-3p. The potential targets of the *DNMT3A* 3′-UTR were mutated (Fig. 3a). Luciferase reporter assays were performed on the *DNMT3A* 3′-UTR and the mutated 3′-UTR in the presence or absence of miR-455-3p overexpression. Co-transfection of *DNMT3A* 3′-UTR luciferase reporter plasmids with miR-455-3p resulted in significantly decreased luciferase activity (44.3%), and the mutation of the miR-455-3p binding sequence significantly diminished this effect (Fig. 3b). The inhibitory rate of miR-455-3p on the wild-type 3′-UTR* DNMT3A* was 44.3%, while it decreased to 16.0% with the mutation of the *DNMT3A* 3′-UTR (Fig. 3b). Together these data indicate that miR-455-3p does modulate *DNMT3A* expression by binding to the 3′-UTR, and that* DNMT3A* is a target of miR-455-3p.

### miR-455-3p inhibits the degenerate process during chondrogenic differentiation of hBMSCs

**Fig. 4:**
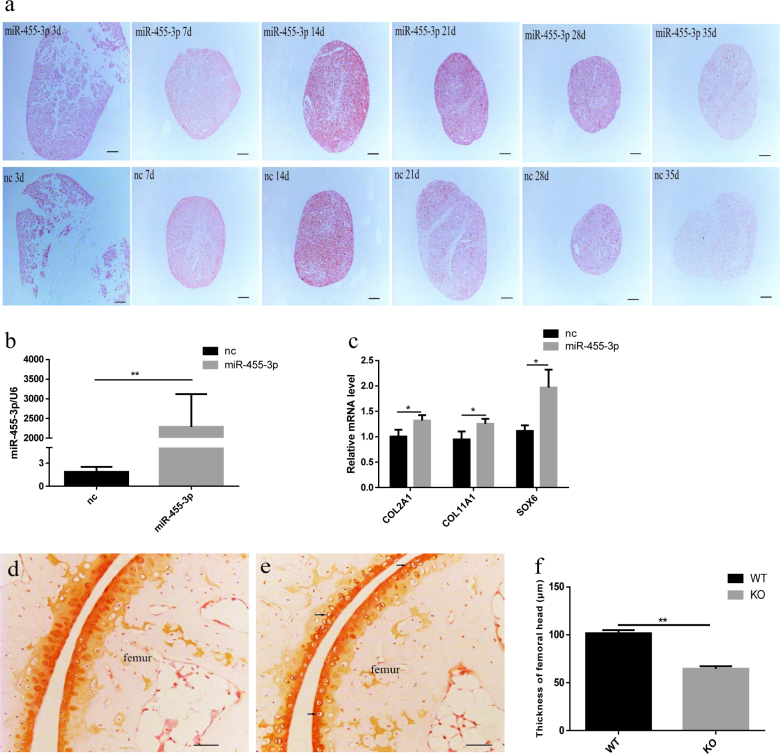
miR-455-3p inhibits cartilage degenerate process in vitro and in vivo. hBMSCs were transfected with miR-455-3p agomir induced to differentiate to chondrocytes in micromass culture. Safranin O was strained for 3, 7, 14, 21, 28, and 35 days of chondrogenic differentiation (**a**). We extracted the RNA on days 28 of the chondrogenic differentiation to detect the expression of miR-455-3p (**b**), COL2A1, COL11A1, and SOX6 (**c**) by RT-PCR. U6 and GAPDH expression levels were measured and used as internal controls for microRNA and mRNA expression, respectively. Wild-type (WT) mice (**d**) and miR-455-3p deletion (KO) mice (**e**) were stained with Safranin O at 6 months of age, scale bar, 100 μm. Average thicknesses of total articular cartilage in femoral head were quantified. miR-455-3p deletion mice showed more hypertrophic chondrocytes (black arrow) and thinner cartilage than WT mice (**f**). Data are presented as means ± SD of the results obtained from triplicate samples. *P < 0.05; **P < 0.001 vs negative controls, respectively

Our previous results showed miR-455-3p expression rapidly increasing from day 3 to day 21, followed by a marked decrease from days 28 to 35 during chondrogenic differentiation. Previous studies also demonstrated that miR-455-3p modulates cartilage development and degeneration^14,15^. Therefore, we overexpressed miR-455-3p in hBMSCs and induced them to differentiate into chondrocytes in micromass culture. Notably, safranin O staining showed that overexpression of miR-455-3p did not affect cartilage development between days 0 and 14, but later inhibited matrix degeneration from days 21 to 35 (Fig. 4a). We then extracted the RNA on day 28 of chondrogenic differentiation to detect the expression of miR-455-3p and cartilage-related mRNAs. Treatment with the miR-455-3p agomir significantly increased the expression of miR-455-3p (Fig. 4b), *COL2A1, COL11A1*, and *SOX6* (Fig. 4c). We also observed the cartilage thickness of hip joint in wild-type mice (Fig. 4d) and miR-455-3p deletion mice at 6 months (Fig. 4e); the miR-455-3p deletion mice exhibited more hypertrophic chondrocytes and thinner cartilage than wild mice (Fig. 4f). These results show that the upregulation effect of the miR-455-3p agomir in hBMSCs can continue for more than 28 days during chondrogenic differentiation. High expression levels of miR-455-3p can inhibit degeneration of the cartilage matrix and increase the expression of cartilage-specific genes, and miR-455-3p deletion mice showed accelerated cartilage degeneration compared with wild mice.

### Significant differential CpG methylation between overexpressed miR-455-3p and control groups in hBMSCs induced to differentiate to chondrocytes

To determine whether miR-455-3p regulates DNA methylation during the chondrogenic differentiation of hMSCs, genome-wide DNA methylation was examined. The DNA methyltransferase *DNMT3A* modifies mammalian genomes by cytosine methylation, an epigenetic mark that is essential for normal development and primarily occurs at CpG dinucleotides^18^. Most CpG-rich regions (CpG islands) overlap with proximal promoters and are linked to gene silencing^19^. We have demonstrated that miR-455-3p inhibits the degeneration process during the chondrogenic differentiation of hBMSCs, and directly targets *DNMT3A* by interaction with the 3′-UTR. Therefore, we concluded that miR-455-3p may regulate the chondrogenic differentiation of hBMSCs by directly targeting *DNMT3A* to affect genomic methylation levels. As described previously, hBMSCs transfected with miR-455-3p agomir or normal controls were induced to differentiate into chondrocytes in micromass culture. We compared the CpG methylation on day 28 of chondrogenic differentiation.

**Fig. 5:**
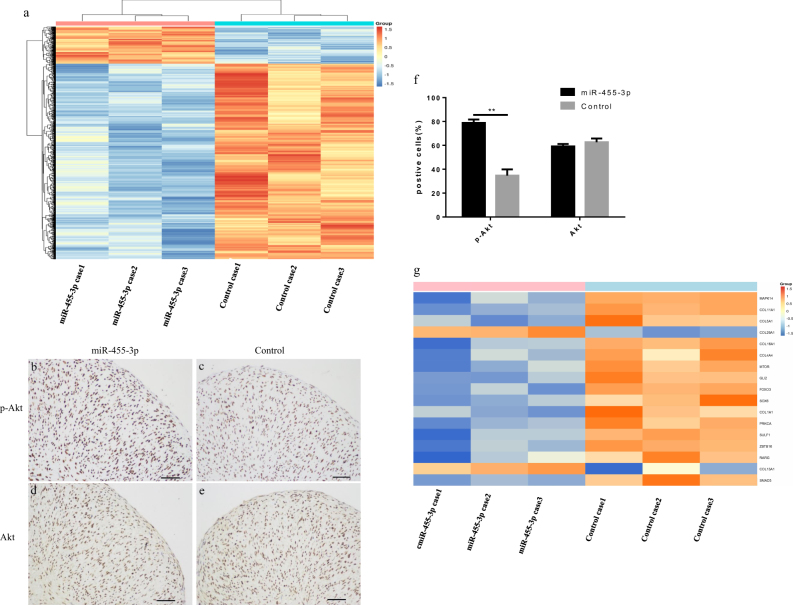
Genome-wide DNA methylation profile during chondrogenic differentiation of hBMSCs. Genome-wide DNA methylation profile during chondrogenic differentiation of hBMSCs. Heatmap of the 1733 significant differentially DNA methylation sites in miR-455-3p group comparing with control group (**a**). hBMSCs were transfected with miR-455-3p agomir or control induced to differentiate to chondrocytes in micromass culture for 28 days, and then immunohistochemistry of p-Akt (**b**, **c**) and Akt (**d**, **e**) were used to evaluated the PI3K/Akt signaling pathway (**f**), scale bar, 100 μm. Data are presented as means ± SD of the results obtained from triplicate samples. *P < 0.05; **P < 0.001 vs negative controls, respectively. CpG methylation of cartilage development related genes (**g**)

We detected 803,869 methylated CpG sites and observed 1733 differentially methylated sites (*P* < 0.01) representing 1023 distinct genes and nearby genomic regions when comparing miR-455-3p groups with control groups (Fig. 5a, Supplementary Figure 3a). Of these 1773 sites, 1491 (838 genes) were hypomethylated and the remaining 282 sites (183 genes) were hypermethylated (Supplementary Table 1). The top 20 CpGs according to difference in methylation levels were listed in Table 1.

**Table 1:** Top differentially hypomethylated and hypermethylated CpG sites

Illumina ID	Associated gene symbol	miR-455-3p *β*	Control *β*	Δ*β*	FDR-P value	CpG location	Location in enhancer
Top hypermethylated sites							
cg03411918		0.85429915	0.654286759	0.20001239	0.002329516		NA
cg26523866		0.732835906	0.567929817	0.16490609	0.001328907		TRUE
cg19695521	HCG18	0.411955285	0.301119676	0.110835609	0.009617246	Body	NA
cg15348274	ABCB9	0.290573575	0.18011309	0.110460485	0.001972283	5′-UTR	NA
cg18633134		0.437928402	0.327541967	0.110386435	0.005910667		NA
cg09916763	NEBL	0.689488949	0.584134654	0.105354296	0.000871015	Body	NA
cg21691809	CLGN	0.133046694	0.033722514	0.09932418	8.33E-05	TSS200	NA
cg24570624	C14orf4	0.331959975	0.234604181	0.097355794	0.00913907	1st Exon	NA
cg19082105	SLC34A1	0.63876831	0.541841261	0.096927048	0.000437297	TSS1500	NA
cg06254044	C11orf65	0.834485137	0.745398323	0.089086815	0.001994917	TSS1500	NA
cg16074241	CSMD3	0.712575497	0.62506252	0.087512977	0.000733914	Body	NA
cg23893009		0.906343875	0.828571632	0.077772244	7.06E-05		NA
cg11705100	ADRA1A	0.900905762	0.823951001	0.076954761	0.008305623	Body	NA
cg14503200	ROR2	0.865238588	0.789787824	0.075450764	0.002193837	Body	NA
cg27269993	ALB	0.886397836	0.81168735	0.074710486	0.003122595	Body	TRUE
cg04177989	LINC00564	0.433394046	0.360589791	0.072804255	0.004139182	TSS1500	NA
cg17793819	ZHX2	0.165543694	0.094208571	0.071335123	0.002716411	TSS200	NA
cg20085197		0.742920417	0.673617976	0.069302441	0.007788236		NA
cg11058939		0.879068917	0.810159701	0.068909215	0.000140968		TRUE
cg12552770	IREB2	0.932863243	0.867463255	0.065399989	0.003824771	Body	NA
Top hypomethylated sites							
cg00449608	GPR45	0.242981495	0.686573289	−0.443591794	0.009516558	TSS1500	NA
cg06198776	PIBF1	0.154669829	0.572247756	−0.417577928	3.35E-05	Body	NA
cg19450830	LINC00548	0.39194816	0.7977394	−0.40579124	0.000220008	TSS1500	TRUE
cg07374695	P2RX7	0.28044041	0.682154337	−0.401713928	0.001304077	Body	NA
cg09627772	RABGAP1L	0.162932352	0.561519876	−0.398587524	0.000884627	Body	NA
cg05007112	GCC2	0.171615788	0.563873804	−0.392258017	0.00172162	Body	NA
cg14708360		0.188967691	0.572140844	−0.383173152	0.000467635		TRUE
cg07475284	HECW2	0.419477422	0.794862044	−0.375384622	0.00024201	5′-UTR	TRUE
cg22130673	ZBTB16	0.37642215	0.748233855	−0.371811705	0.001092287	Body	TRUE
cg00608183		0.227348067	0.598587288	−0.371239221	0.000506866		NA
cg00979515		0.222629852	0.593228804	−0.370598952	7.97E-05		NA
cg02390981	KLC1	0.408629455	0.77894639	−0.370316935	0.000404981	Body	NA
cg22602094	ADAMTS16	0.163089519	0.524240086	−0.361150567	0.004151807	Body	NA
cg13201036	EPN2	0.095664948	0.454884995	−0.359220047	0.000512488	Body	NA
cg11769597		0.145825087	0.502098007	−0.35627292	0.000452804		NA
c g05559888		0.254035348	0.608152643	−0.354117295	0.003276138		NA
cg20224091		0.11584416	0.469822353	−0.353978192	1.35E-05		NA
cg21542303	NFKB1	0.188709635	0.541788057	−0.353078422	0.002441491	Body	NA
cg23295942		0.228562019	0.580751528	−0.352189509	0.005733339		NA
cg13280925	SEC63	0.411386516	0.760081635	−0.348695119	0.002720771	Body	NA

Δ*β* difference in methylation value between sample groups (*β*_miR-455-3p_−*β*_control_), *FDR* false discovery rate, *NA* not applicable, *TSS 1500* within 1500 bp of transcription start site, 5′-UTR 5’-untranslated region.

### Gene ontology (GO) and pathway analysis of differentially methylated CpG sites

GO categories included biological processes, cellular components, and molecular functions, for which cellular component organization, cell junctions, and protein binding were the most diverse terms, respectively (Supplementary Figure 3b), while the PI3K-Akt signaling pathway was the most discrepant pathway (Supplementary Figure 3c). We analyzed the hypomethylation and hypermethylation sites, respectively. In the GO analysis, the genes related to positive regulation of NF-kB transcription factor activity were the most differentially upregulated by methylation (*P* = 1.35 × 10^−6^, FDR = 9.2 × 10^−5^), while the genes related to signal transduction were most differentially downregulated by methylation (*P* = 1.39 × 10^−14^, FDR = 3.33 × 10^−10^). The top five terms of GO analysis according to difference in methylation levels are listed in Table 2. Notably, the genes related to collagen biosynthesis (*P* = 1.14 × 10^−^^7^, FDR = 3.42 × 10^−^^5^) and positive regulation of cartilage development (*P* = 0.005, FDR = 0.09) are hypomethylated. In the pathway analysis, the endocytosis pathway was the most hypermethylated (*P* = 0.04, FDR = 1), while the PI3K-Akt signaling pathway is the most hypomethylated (*P* = 1.20 × 10^−^^19^, FDR = 1.53 × 10^−^7). The ECM−receptor interaction was also hypomethylated (*P* = 0.002, FDR = 0.015), as shown in Table 3. To confirm the DNA methylation analysis results, we overexpressed miR-455-3p in hBMSCs and induced them to differentiate to chondrocytes in micromass culture for 28 days, and immunohistochemistry of Akt and p-Akt were used to evaluate the PI3K/Akt signaling pathway. Overexpression of miR-455-3p upregulated p-Akt expression (Fig. 5b−f).

**Table 2:** Gene ontology analysis

Pathway	No. of DM genes	*P*	FDR
**Hyper-methylated**			
Positive regulation of NF-kB transcription factor activity	7	1.35E-06	0.000920046
Transmembrane transport	12	9.41E-06	0.003198452
Embryonic skeletal system development	4	1.99E-05	0.004507739
Anterior/posterior pattern specification	5	0.000131002	0.022270268
Multicellular organismal homeostasis	2	0.000195932	0.026646776
**Hypo-methylated**			
Signal transduction	63	1.39E-13	3.33E-10
Positive regulation of transcription from RNA polymerase II promoter	46	5.75E-11	6.87E-08
Small molecule metabolic process	67	4.21E-10	3.35E-07
Negative regulation of transcription from RNA polymerase II promoter	36	9.78E-10	5.85E-07
Intracellular signal transduction	25	6.94E-09	3.32E-06

*DM* differentially methylated

**Table 3:** Differentially methylated canonical pathways between miR-455-3p and control groups

Pathway	No. of DM genes	*P*	FDR
**Hyper-methylated**			
Endocytosis	4	0.039186726	1
**Hypo-methylated**			
PI3K-Akt signaling pathway	29	1.20652E-09	1.53573E-07
Pathways in cancer	28	1.40249E-09	1.53573E-07
Metabolic pathways	47	0.000117961	0.001987193
Wnt signaling pathway	11	0.000824249	0.009500549
ECM−receptor interaction	8	0.001821074	0.015339048

### Overexpression of miR-455-3p reduced the methylation of cartilage development-related genes

Next, we analyzed the CpG methylation of cartilage development-related genes such as *COL5A1, COL11A1, SMAD3, SOX6, FOXO3A, MTOR*. Most of these genes are hypomethylated, except for COL13A1 and COL29A1 (Fig. 5g). Notably, most of these hypomethylated genes are related to the PI3K-Akt signaling pathway and/or the ECM−receptor interaction pathway. For example, collagen type V alpha 1 (COL5A1), a gene that promotes the anabolism of cartilage^20^, was hypomethylated with the overexpression of miR-455-3p, and is also related to the PI3K-Akt signaling pathway and the ECM−receptor interaction pathway. These data suggest that miR-455-3p inhibits the degeneration process of chondrogenic differentiation by activating canonical pathways such as the PI3K-Akt signaling pathway through the reduction of its methylation levels.

## Discussion

In this study, we first identified the co-regulating activity of *DNMT3A* and miR-455-3p in DNA methylation during the degenerate stage of chondrogenic differentiation. Our results demonstrated that miR-455-3p regulated the expression of *DNMT3A*, and *DNMT3A* also modulated miR-455-3p expression in vitro. The in vivo study showed high *DNMT3A* expression levels in miR-455-3p deletion mice and low levels in wild-type mice. We also observed thinner cartilage thickness in miR-455-3p deletion mice at 6 months of age than we did in wild-type mice. Additionally, a luciferase reporter assay demonstrated that miR-455-3p regulates *DNMT3A* expression by binding to the *DNMT3A* 3′-UTR. Overexpression of miR-455-3p inhibits the degenerate process during chondrogenic differentiation by regulating DNA methylation of cartilage development-related genes and pathways.

In recent years, miRNAs have drawn increasing interest due to their regulation of a wide variety of genes, including the cartilage-specific genes^21,22,23^. MiR-455 is located in an intronic region of *COL27A1*^24^, Swingler et al. reported that miR-455-3p is highly expressed during the chondrogenesis of ATDC5 cells, and that it regulates TGFβ signaling and suppresses the Smad2/3 pathway^25^. We also identified eight miRNAs with over twofold increases in regulation during chondrogenic differentiation of hADSCs, such as miR-320c, miR-92a-3p, and miR-455-3p^13^. Recent studies have shown that miR-455-3p functions as suppressors of hypoxia signaling^24^ and gastric cancer^26^. Our previous studies demonstrated that miR-455-3p regulated hMSC chondrogenic differentiation by targeting *RUNX2*^15^, *HDAC2*, and *HDAC8*^14^.

Moreover, our results also demonstrated that miR-455-3p targets the 3′-UTR of *DNMT3A* mRNA and downregulates its expression. *DNMT3A* is a de novo DNA methyltransferase that regulates DNA methylation, thus implicating it in diverse biological processes. MiR-101 affects lung cancer progression by targeting *DNMT3A* to regulate the PTEN/AKT signaling pathway^27^. MiR-200b-3p inhibits the secretion of MMPs and promotes the synthesis of collagen type II in OA chondrocytes by inhibiting the expression of *DNMT3A*^28^. In a previous study, knockdown of *DNMT3A* upregulated the transcription of arterial marker genes *E2F1*, thereby promoting the arterial specific differentiation of hMSCs^29^. Our study reveals that overexpression of miR-455-3p inhibits matrix degeneration and increases the expression of the cartilage-specific genes *COL2A1, COL11A1*, and *SOX6* during the degenerate stage of chondrogenic differentiation (days 21−35).

It has long been recognized that DNA methylation plays an important role in the occurrence and development of osteoarthritis. The differential DNA methylation of genes including *AGO2, TGFβ3*, and *HDAC4* were observed when comparing the cartilage intact with subchondral bone and overlying cartilage in patients with hip osteoarthritis^30^. Another study suggested that DNA methylation of cartilage-related genes and pathways changes in OA patients, especially at the late stage of OA^31^. Recent studies have shown that DNA methylation has emerged as a crucial regulator of chondrocyte dedifferentiation. Treatment with the DNA methylation inhibitor 5-azacytidine reverses the chondrocyte dedifferentiation and increases *SOX-9* expression^32^. Our study demonstrated that miR-455-3p plays a role similar to 5-azacytidine in repressing *DNMT3A* expression and inhibiting DNA methylation. miR-455-3p overexpression downregulates the DNA methylation of cartilage development-related genes such as *SOX6* and *Smad3*. Both upregulation of *SOX6*^33^ and *Smad3*^34^ promotes chondrogenic differentiation of hMSCs.

The PI3K-Akt signaling pathway is essential for the process of differentiation^35^. Activation of the pathway enhance chondrocyte proliferation and inhibit hypertrophic differentiation^36,37^, and its inhibition increases apoptosis^38^. GO analysis of differentially methylated sites revealed the PI3K-Akt signaling pathway as the most hypomethylated pathway. These data demonstrate that miR-455-3p can inhibit the expression of *DNMT3A* to regulate DNA methylation of cartilage-specific genes, thus regulating pathways such as the PI3K-Akt signaling pathway and ultimately inhibiting the cartilage degeneration during chondrogenic differentiation.

In summary, this study demonstrates that miR-455-3p and *DNMT3A* co-regulate chondrogenic differentiation of hMSCs by modulating DNA methylation. The increased expression of miR-455-3p and decreased expression of *DNMT3A* upregulate cartilage-specific genes and pathways, thereby attenuating cartilage degeneration during chondrogenic differentiation. To the best of our knowledge, this study is the first to demonstrate the co-regulation between *DNMT3A* and miR-455-3p in vivo and the chondrogenic differentiation of hBMSCs in vitro. These results not only reveal the important role of DNA methylation in chondrogenic differentiation, but also demonstrate that miR-455-3p can regulate this process by affecting DNA methylation. Overexpression of miR-455-3p and the use of a DNA methylation inhibitor could optimize the process of chondrogenic differentiation.

## Materials and methods

All procedures were approved by the ethical committee of the First Affiliated Hospital of Sun Yat-Sen University (IRB: 2014C-028) and the Helsinki Declaration (2000). All volunteers provided written informed consent.

### Articular chondrocyte isolation and culture

Human normal articular cartilage samples were obtained from five patients (one male and four females; mean ± SD age, 65.4 ± 4.0 years) who had no history of OA or rheumatoid arthritis and who underwent hemiarthroplasty or total hip arthroplasty because of femoral neck fractures. Informed consent was obtained from all patients. The cartilages were dissected away from the subchondral bone and then digested by 4 mg/mL protease and 0.25 mg/mL collagenase P as described previously^16^. Cells were cultured in DMEM/Nutrient Mixture F-12 (Gibco Life Technology Grand Island, NY, USA) containing 5% fetal bovine serum (FBS; Gibco Life Technology), 100 IU/mL penicillin, and 100 μg/mL streptomycin (Gibco Life Technology). The chondrocytes were used in experiments within 3–7 days and without passaging to avoid dedifferentiation.

### Human mesenchymal stem cell isolation, culture, and chondrogenic differentiation

Bone marrow samples were obtained from three normal human donors (one male and two females; mean ± SD age, 25.3 ± 3.8 years), and hBMSCs were isolated as described previously^16^. Cells were cultured in Alpha-modified Eagle’s medium (a-MEM) (Gibco Life Technology Grand Island, NY, USA) supplemented with 10% FBS, 100 IU/mL penicillin, and 100 μg/mL streptomycin. The hMSCs were induced to differentiate into chondrocytes in micromass culture within three passages as described previously^16^. Briefly, hBMSCs were resuspended in a-MEM supplemented with 10% FBS at a specified density of 10^5^ cells/μL of media, and 12.5 μL of the suspended cells were dotted on the center of each well of 24-well plates and then cultured in the 37 °C incubator for 1 h to stimulate the adherence of the cells to the plate. Next, the cells were cultured with chondrogenic induction medium (human mesenchymal stem cell chondrogenic differentiation basal medium 97 mL, TGF-β3 1 mL, dexamethasone 10 μL, ascorbate 300 μL, ITS (insulin, transferrin, selenium) supplement 1 mL, sodium pyruvate 100 μL, proline 100 μL) (Cyagen, Guangzhou, China), or incomplete chondrogenic induction medium without TGF-β3 as control. At indicated time points, the cells were subjected to RNA extraction or stained with safranin O or immunohistochemistry.

### Animals and in situ hybridization

The mmu-miR-455-3p global knockout mice were generated by a transcription activator-like effector nuclease (TALEN) system. Briefly, we use 1 μm of linearized plasmid as a template for an in vitro transcription reaction. Next, synthesized RNA was treated with Poly(A) Tailing Kit (Life Technologies) to add poly(A) tail. Microinjection of a mixture of RNAs into cytoplasm of one-cell stage embryo, and then two-cell stage embryos were transferred into pseudopregnant C57BL/6 female mice. For genotyping, DNA sequencing analysis was performed after PCR. The wild-type (miR-455-3p+/+), heterozygous (miR-455-3p+/−), and homozygous (miR-455-3p−/−) mice were obtained through the mating of heterozygous male and female mice. In our study, we compared the wild-type mice and homozygous mice.

The knee joint of miR-455-3p deletion mice and wild-type mice were harvested at 6 months and fixed by incubation in DEPC-treated 10% formalin at 4 °C overnight. Tissues were subsequently dehydrated with a graded series of ethanol, embedded in paraffin, and cut into 5-μm-thick sections. Sections were subjected to in situ hybridization analysis using the miR- 455-3p-specific probe (Servicebio, Wuhan, China), or treated with PBS instead of the miR-455-3p probe as negative control.

### Transfection

The normal chondrocytes were transfected with miR-455-3p mimic or inhibitor (RiboBio, Guangzhou, China) at a concentration of 50 nM; they were also transfected with DNMT3A-siRNA or NC (RiboBio, Guangzhou, China). Lipofectamine^®^ 2000 Transfection Reagent (Gibco Life Technologies) was used to transfect cells according to the manufacturer’s instructions. Cells were then harvested after 48 h for quantitative real-time reverse transcription-polymerase chain reaction (qRT-PCR), or after 72 h for western blot analysis.

### RT-PCR analysis, western blot, and immunohistochemistry

Total cellular RNA was isolated using miRNeasy Mini Kit (Qiagen, Venlo, Netherlands). Next, cDNA was synthesized from mRNA and miRNA by using PrimeScript™ RT Master Mix (Takara, Shiga, Japan) and Mir-X™ miRNA First-Strand Synthesis Kit (Clontech Laboratories, Inc., Mountain View, CA, USA), respectively. qPCR of miR-455-3p and target mRNAs were performed using SYBR^®^ Premix Ex Taq™ II (Takara) and KOD SYBR^®^ qPCR Mix(Toyobo), according to the manufacturer’s instructions, respectively. Transcript levels were normalized to that of the housekeeping gene glyceraldehyde 3-phosphate dehydrogenase (GAPDH; for mRNA) or the small U6 RNA (for miRNA). The specific primers used for these analyses are listed in Supplementary Table 2. Gene expression was calculated using the 2^−ΔΔCt^ method, and each experiment was performed in triplicate. Western blotting analysis was performed as described previously^16^. Briefly, total proteins were isolated from normal or OA chondrocytes by RIPA buffer (Beyotime Biotechnology, Beijing, China) containing protease inhibitors (Abcam) to obtain whole cell extracts. Membranes were incubated with primary antibodies (1:1000 dilution) against DNMT3A (D23G1, Cell Signaling Technology), SOX9 (D8G8H, Cell Signaling Technology), and H3K4me3 (C42D8, Cell Signaling Technology). GAPDH (EPR16891, Abcam) was utilized as an internal control. Immunohistochemical analysis was carried out as described previously^16^. Sections were treated with 3% normal goat serum for 1 h and incubated with antibodies specific to DNMT3A (D23G1, Cell Signaling Technology), p-Akt (D9E, Cell Signaling Technology), and Akt(C67E7, Cell Signaling Technology). Sections were also stained with safranin O.

### Luciferase constructs and reporter assay

The DNMT3A sequences of DNMT3A 3′-UTR were amplified by polymerase chain reaction (PCR) using the following primers: forward 5′-ATAGGCCGGCATAGACGCGTAGCGGT CTAAAGCATCCC-3′ and reverse 3′-AAAGATCCTTTATTAAGCTTTTATTACGGTGC TTTTCCATTT-5′. The seed sequences were mutated with the following primers: forward 5′-TTATTACGGTGCTTTTCCATTTTTCTTTTTGCACAAAACTTTCGTGTTTATTCAAG TGTTCTCCAGCA-3′ and reverse 3′-ACACCGTCGAACAATCACTCTTGCATT GATTTC-5′. The amplified DNA sequences were inserted into the pmiR-RB-REPORT™ Vector (OBIO, Shanghai, China) to generate DNMT3A 3′-UTR or mutated DNMT3A 3′-UTR luciferase vectors. For the dual luciferase assay, 1.2 × 10^4^ cells (HEK293T) in a 96-well plate were transfected with 50 nM hsa-miR-455-3p or mimic NC (RiboBio). The cells were then co-transfected with 2 μg/mL of vector with the wild-type or mutant DNMT3A 3′-UTR. Luciferase activity was measured 48 h after transfection by the Dual-Luciferase^®^ Reporter Assay System (Promega, Madison, WI, USA) according to the manufacturer’s instructions. Luciferase assays were performed in three independent experiments.

### Differential methylation analysis and gene ontology analyses

Genomic DNA was extracted using QIAamp DNA Mini Kit (QIAGEN, Tokyo, Japan). After the quality and quantity identification by agarose gel electrophoresis Spectrophotometer (SpectraMax Plate Reader, Molecular Devices, Sunnyvale, CA), DNA (1 μg) was bisulfite treated using an EZ DNA methylation kit (Zymo Research, Irvine, CA), and genome-wide DNA methylation was assessed using an Illumina Human MethylationEPIC microarray (Illumina, San Diego, CA, USA), which analyzes the methylation status of >850,000 methylation sites throughout the genome. GenomeStudio Methylation Module v1.8 (Illumina) was used to analyze the differential methylation sites, according to the manufacturer’s instructions. The percentage of methylated cytosine at each CpG site was calculated as the ratio of methylated probe signal to total locus signal intensity (*β*: 0−1) by GenomeStudio, and then average *β* values were compared. We defined differentially methylated CpG sites as having a *P* value less than or equal to 0.01 after adjusting for multiple testing using GenomeStudio. The GO Database was used for GO analysis. Total genes were treated as the background genes, a list of differential genes was selected, and then a hypergeometric distribution calculation was used to test whether the genes in the list were significantly enriched.

### Statistical analysis

Statistical analyses were performed using SPSS 19.0 software (IBM Corporation, Armonk, NY, USA). Data are presented as mean ± standard deviation (SD) of the results of at least three independent experiments. Student’s *t*-test was used to identify differences between groups. One-way analysis of variance and Kruskal–Wallis tests were used for performing multiple group comparisons. *P* < 0.05 was considered statistically significant for all the tests.

## Electronic supplementary material

Supplementary Figure 1

Supplementary Figure 2

Supplementary Figure 3

Supplementary Table 1

Supplementary Table 2
