# Supplementary material for: MiR-455-3p inhibits the degenerate process of chondrogenic differentiation through modification of DNA methylation
Source: Cell Death Dis. 2018 May 10;9(5):537. doi: 10.1038/s41419-018-0565-2 (PMC5945650; doi:10.1038/s41419-018-0565-2)
Supplement: Supplementary file 1 — Supplementary Figure 1 [file 41419_2018_565_MOESM1_ESM.pdf]

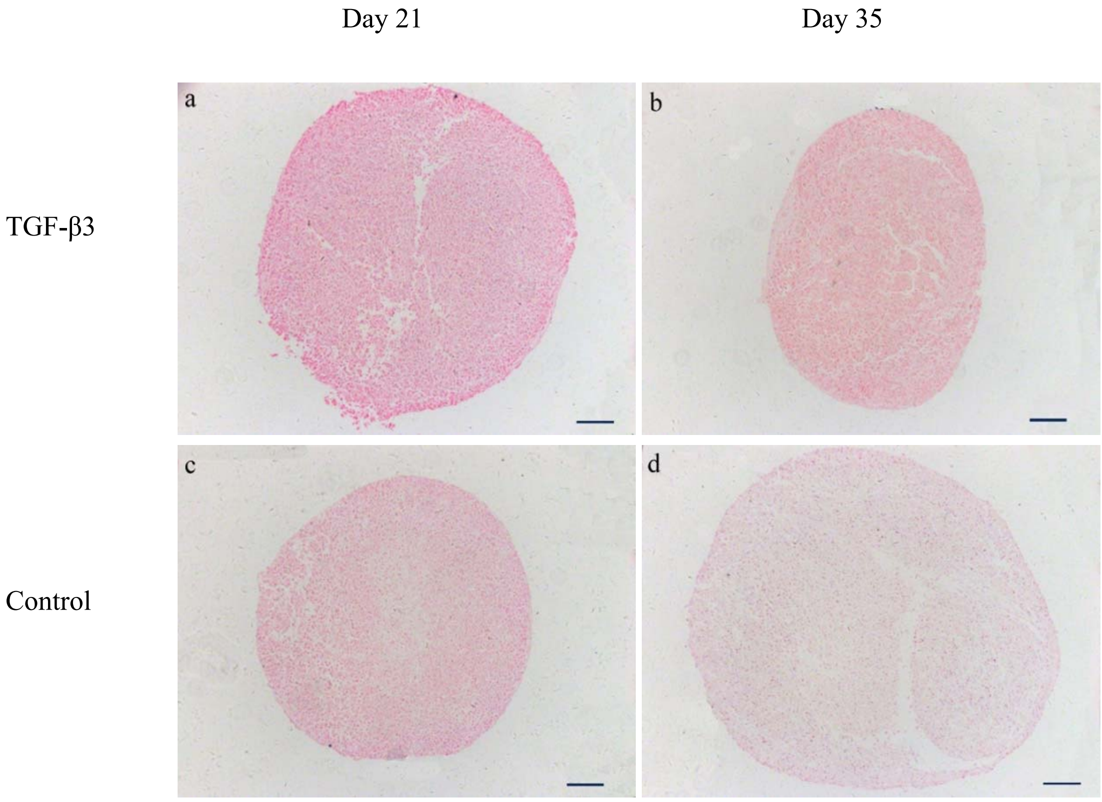

Supplementary Figure 1. Safranin O staining of hBMSCs chondrogenic differentiation at day 21 and 35. hBMSCs were cultured with chondrogenic induction medium (a, b) or incomplete chondrogenic induction medium without TGF-β3 (c, d). Safranin O staining was performed at day 21 (a, c) and 35(b, d). The experiment was performed in triplicate and a representative image is shown.
