# Supplementary material for: MiR-455-3p inhibits the degenerate process of chondrogenic differentiation through modification of DNA methylation
Source: Cell Death Dis. 2018 May 10;9(5):537. doi: 10.1038/s41419-018-0565-2 (PMC5945650; doi:10.1038/s41419-018-0565-2)
Supplement: Supplementary file 2 — Supplementary Figure 2 [file 41419_2018_565_MOESM2_ESM.pdf]

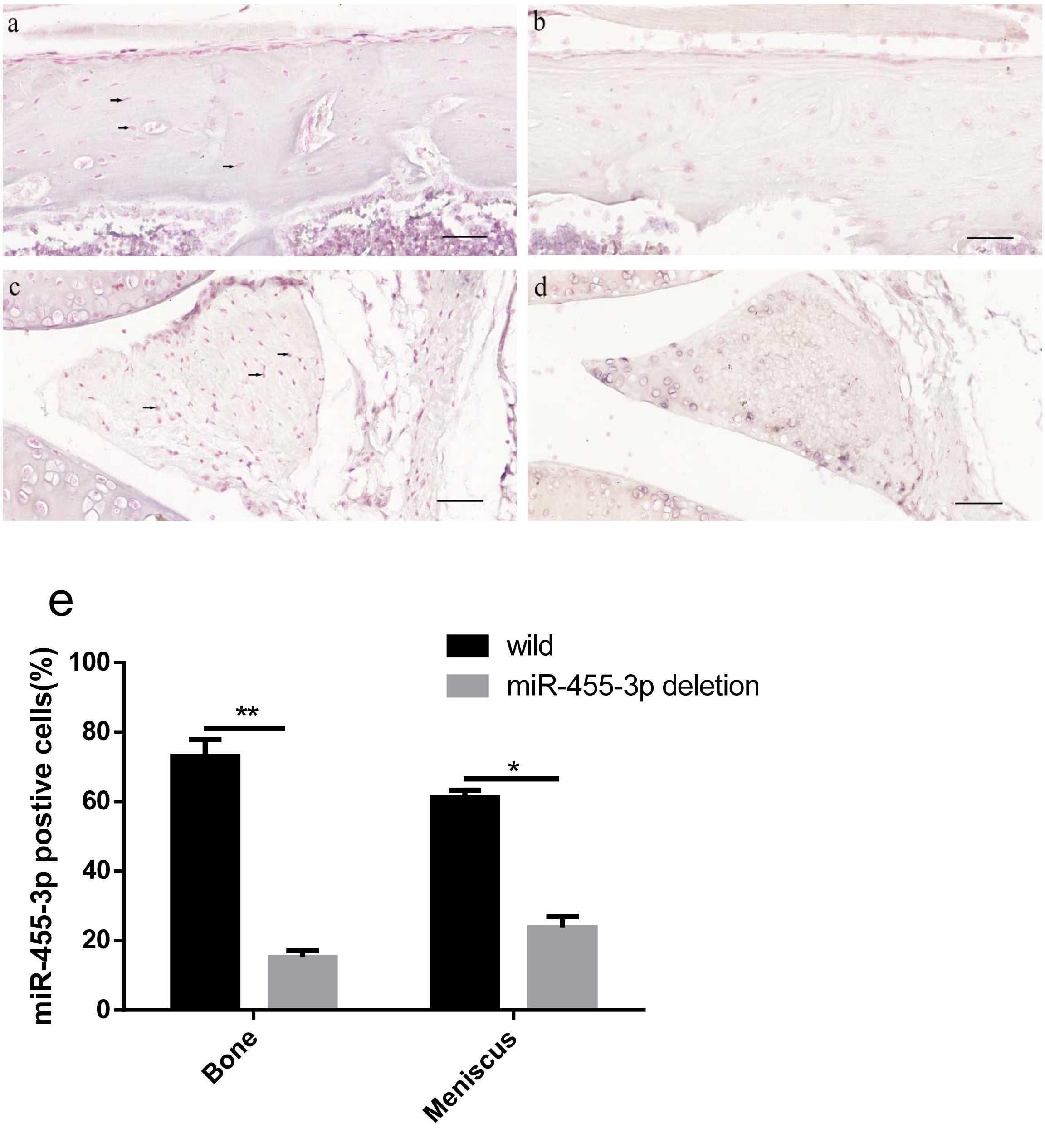

Supplementary Figure 2. The expression of miR-455-3p in bone and meniscus of wild mice and miR-455-3p deletion mice. Femoral cortical (a, b) and anteriomedialis meniscus (c, d) situ hybridization showed the miR-455-3p expression in miR-455-3p deletion mice (b, d) was repressed compared with wild mice (a, c, e), scale bar, 100μm. The experiment was performed in triplicate and a representative image is shown.
