# Supplementary material for: MiR-455-3p inhibits the degenerate process of chondrogenic differentiation through modification of DNA methylation
Source: Cell Death Dis. 2018 May 10;9(5):537. doi: 10.1038/s41419-018-0565-2 (PMC5945650; doi:10.1038/s41419-018-0565-2)
Supplement: Supplementary file 3 — Supplementary Figure 3 [file 41419_2018_565_MOESM3_ESM.pdf]

a

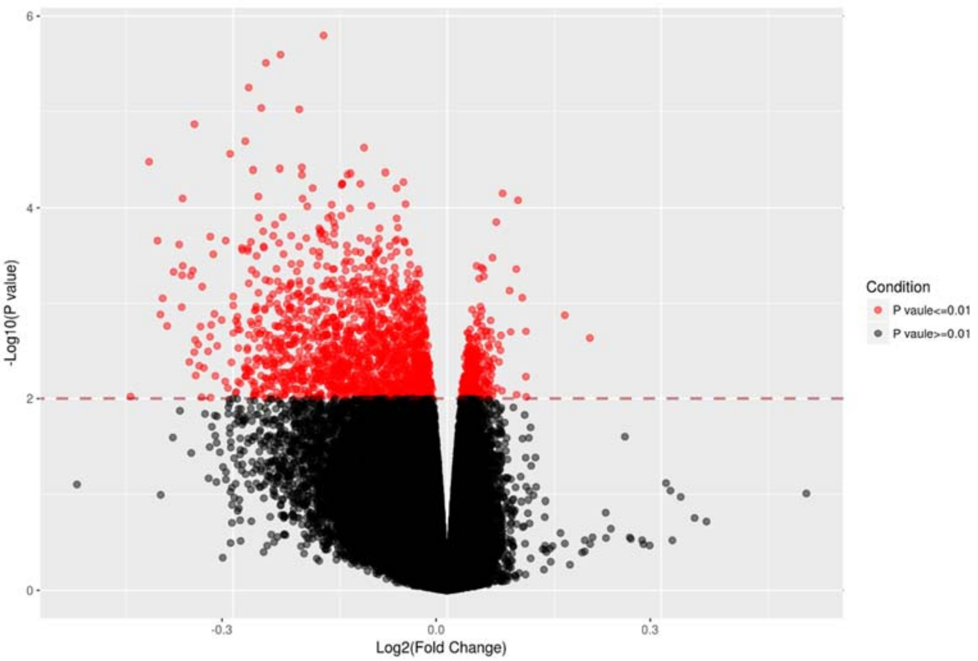

-Log10(P value)
Log2(Fold Change)
Condition
P vaule<=0.01
P vaule>=0.01

b

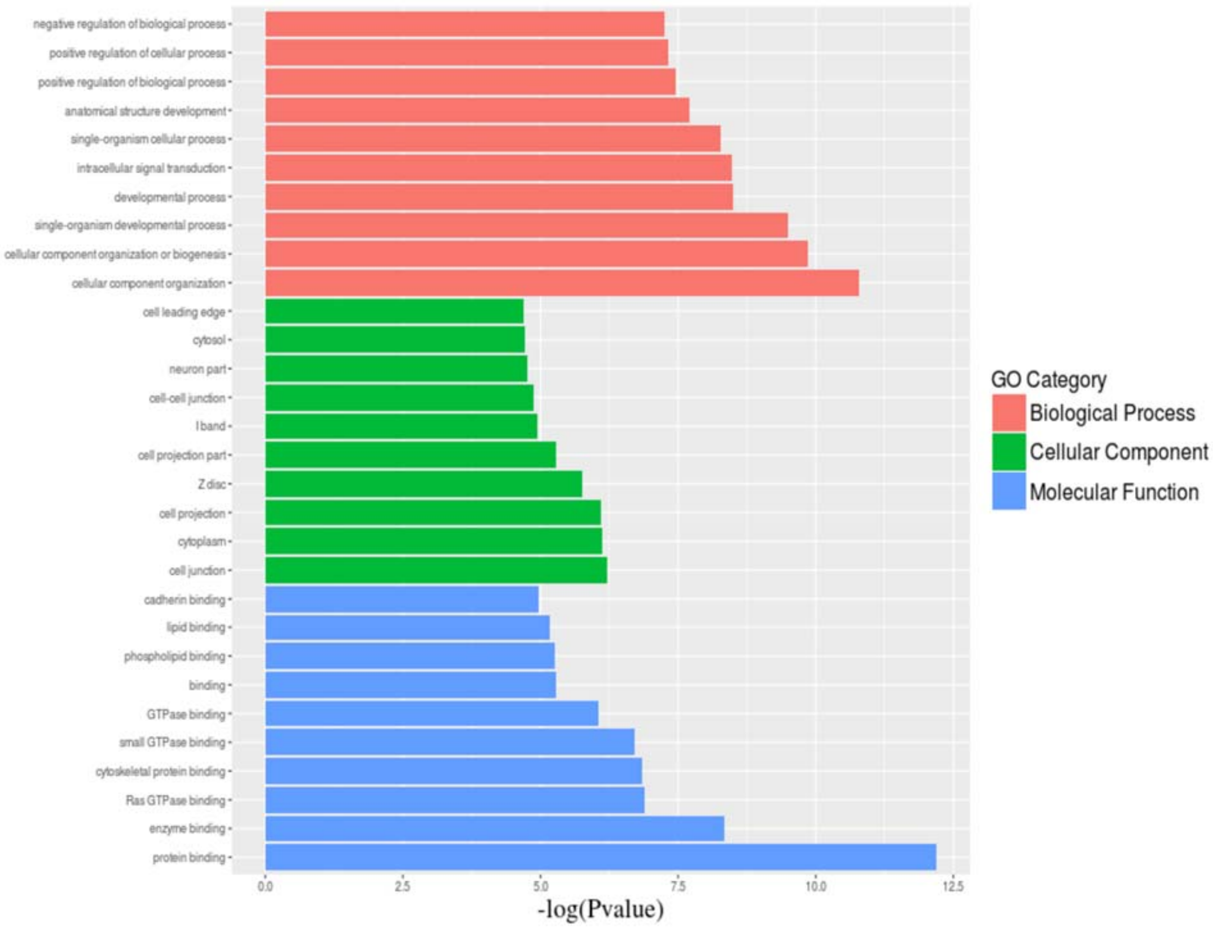

negative regulation of biological process
positive regulation of cellular process
positive regulation of biological process
anatomical structure development
single-organism cellular process
intracellular signal transduction
developmental process
single-organism developmental process
cellular component organization or biogenesis
cellular component organization
cell leading edge
cytosol
neuron part
cell-cell junction
I band
cell projection part
Z disc
cell projection
cytoplasm
cell junction
cadherin binding
lipid binding
phospholipid binding
binding
GTPase binding
small GTPase binding
cytoskeletal protein binding
Ras GTPase binding
enzyme binding
protein binding
-log(Pvalue)
GO Category
Biological Process
Cellular Component
Molecular Function

c

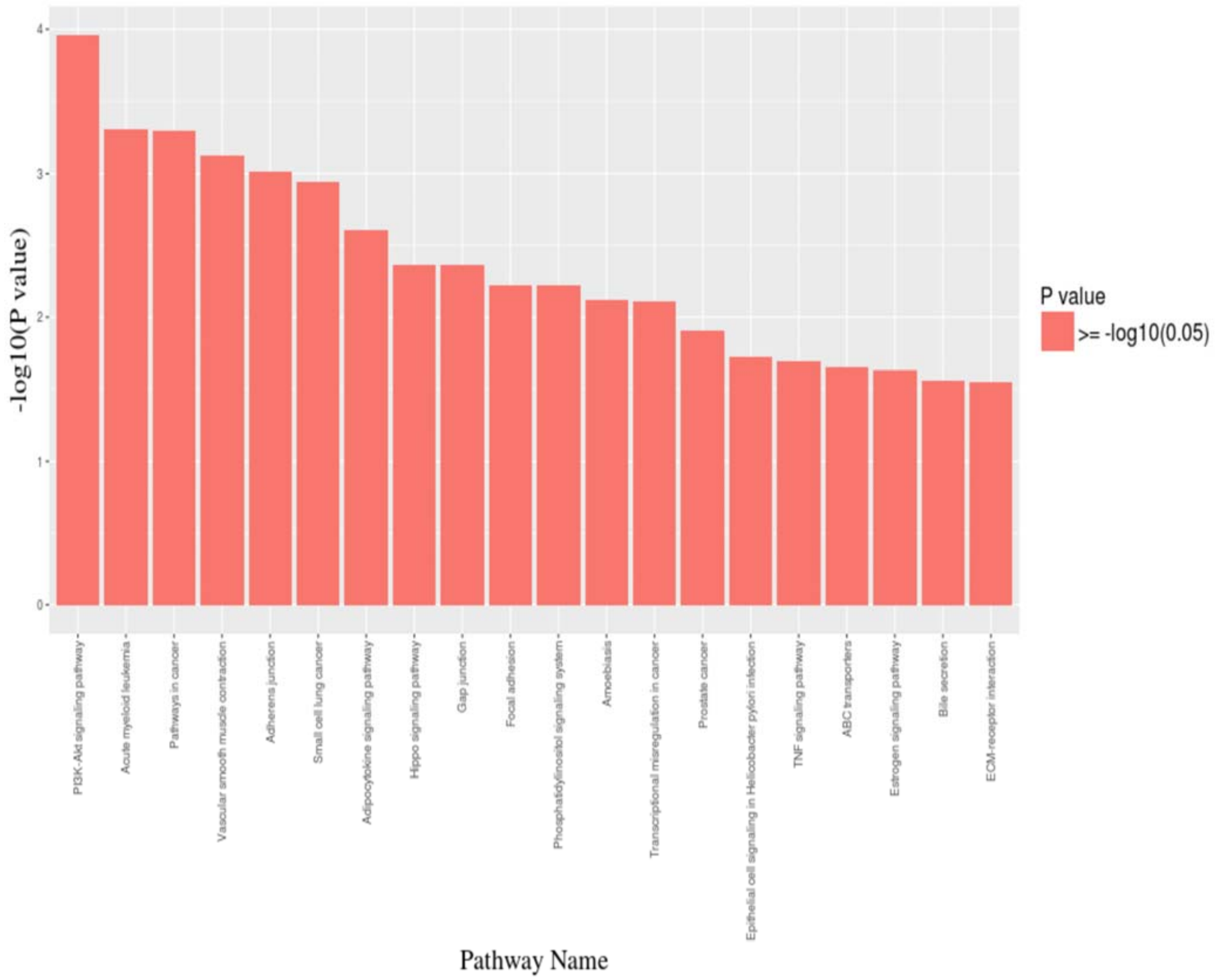

Supplementary Figure 3. Volcano Plot and pathway analysis of the differentially DNA methylation sites. Volcano Plot of the differentially DNA methylation sites. The dots on right side represent hypermethylation of miR-455-3p group comparing with control group, while the left side represent hypomethylation. The DNA methylation sites above the red line indicated more than 2.0 folds change(a). GO and pathway analysis of the differential DNA methylation genes (b, c).
