# Supplementary material for: MiR-455-3p inhibits the degenerate process of chondrogenic differentiation through modification of DNA methylation
Source: Cell Death Dis. 2018 May 10;9(5):537. doi: 10.1038/s41419-018-0565-2 (PMC5945650; doi:10.1038/s41419-018-0565-2)
Supplement: Supplementary file 4 — Supplementary Table 1 [file 41419_2018_565_MOESM4_ESM.docx]

**Supplementary Table 1:** Primers for quantitative real-time polymerase chain reaction (qRT-PCR)

| Gene |  | Primer sequence (5’-3’) |
| --- | --- | --- |
| hsa-miR-455-3p | F | GCGTCCATGGGCATATACAC |
| hsa-U6 | F | CTCGCTTCGGCAGCACA |
| hsa-U6 | R | AACGCTTCACGAATTTGCGT |
| Hsa-DNMT3A | F | GTCATGTGGTTCGGAGACGG |
| Hsa-DNMT3A | R | AGTGTCACTCTCATCGCTGTC |
| hsa-SOX6 | F | AGGGAGTCTTGCCGATGTG |
| hsa-SOX6 | R | CAGGCTCTCAGGTGTACCTTTA |
| hsa-SOX9 | F | GGAGATGAAATCTGTTCTGGGAATG |
| hsa-SOX9 | R | TTGAAGGTTAACTGCTGGTGTTCTG |
| hsa-RUNX2 | F | CACTGGCGCTGCAACAAGA |
| hsa-RUNX2 | R | CATTCCGGAGCTCAGCAGAATAA |
| hsa-aggrecan | F | GATGTTCCCTGCAATTACCACCTC |
| hsa-aggrecan | R | TGATCTCATACCGGTCCTTCTTCTG |
| hsa-COL2A1 | F | GCACCTGCAGAGACCTGAAAC |
| hsa-COL2A1 | R | GCAAGTCTCGCCAGTCTCCA |
| hsa-COL11A1 | F | ACCCTCGCATTGACCTTCC |
| hsa-COL11A1 | R | TTTGTGCAAAATCCCGTTGTTT |
| hsa-GAPDH | F | GCACCGTCAAGGCTGAGAAC |
| hsa-GAPDH | R | TGGTGAAGACGCCAGTGGA |
